# Association Between Dietary Iron Intake and Serum Ferritin and Severe Headache or Migraine

**DOI:** 10.3389/fnut.2021.685564

**Published:** 2021-07-06

**Authors:** Shu-Han Meng, Hai-Bo Zhou, Xin Li, Ming-Xue Wang, Li-Xin Kang, Jin-Ming Fu, Xia Li, Xue-Ting Li, Ya-Shuang Zhao

**Affiliations:** Department of Epidemiology, College of Public Health, Harbin Medical University, Harbin, China

**Keywords:** dietary iron, serum ferritin, migraine, NHANES, restricted cubic spline

## Abstract

**Background:** Dietary iron intake and serum ferritin in relation to severe headache or migraine remain largely unknown. Therefore, we investigated the associations between dietary iron intake and serum ferritin with severe headache or migraine among American adults.

**Methods:** This cross-sectional study included 7,880 adults (≥20 years) from the National Health and Nutrition Examination Surveys (NHANES) of America from 1999 to 2004. We performed multivariable logistic regression and restricted cubic spline (RCS) regression to assess the association of dietary iron and serum ferritin with severe headache or migraine.

**Results:** Most women aged 20–50 years consumed less dietary iron than their recommended dietary allowances. Dietary iron intake was inversely associated with severe headache or migraine in women aged 20–50 years. For women over 50 years, serum ferritin was negatively associated with severe headache or migraine. For men, there was no significant relationship between dietary iron and serum ferritin, and severe headache or migraine.

**Conclusions:** Dietary iron intake has different effects on migraine in women of different ages, and this different effect may be due to age-related menstrual changes. Women aged 20–50 years should have a higher awareness of RDA and increase their dietary iron intake if needed, which may play an important role in preventing severe headache or migraine. Higher serum ferritin levels in women aged 50 and above may have a protective effect against migraine.

## Introduction

Severe headache and migraine are common neurological disorders that can seriously affect people's daily activities and cause substantial burdens to individuals and society (1). In the US, the prevalence of migraine was about 18.2% in females and 6.5% in males and was highest among people aged 25–55 years (2). However, the causes of migraine have not been fully elucidated.

Iron is an essential trace element that regulates cerebral function (3, 4), cell function (5, 6), energy metabolism (7, 8), and neurotransmitter synthesis (9, 10). Headache or migraine in women is usually associated with menstruation, and the incidence of migraine decreases with natural menopause (11, 12). It is a well-known fact that iron is closely related to menstruation, during which iron is lost (13). Furthermore, patients with iron deficiency or iron-deficiency anemia had a high frequency of migraine (14, 15). Therefore, we hypothesized that iron deficiency might be associated with severe headache or migraine.

Dietary iron is the primary source of iron in the body. However, to our knowledge, no other previous studies have directly investigated the relationship between dietary iron intake and severe headache or migraine. Ferritin, an important biomarker of body iron stores (16), deserves attention for its relationship with migraines. An observational study found lower serum ferritin levels in woman migraineurs (17), suggesting a role for dietary iron intake in the management of migraine. Dopamine plays a role in the pathogenesis of migraine, and iron is an essential trace element for the synthesis of dopamine (18–20). Studies also suggest that iron deficiency could lead to dopaminergic neurodegeneration (21–23).

We hypothesized that dietary iron and serum ferritin would be inversely associated with severe headache or migraine. For the first time, we investigated whether dietary iron intake is indeed associated with migraine to test our hypothesis. We also investigated whether serum ferritin was negatively associated with severe headache or migraine.

## Methods

### Study Populations

The National Health and Nutrition Examination Surveys (NHANES) are cross-sectional surveys administered by the National Center for Health Statistics of the Centers for Disease Control and Prevention (CDC) to assess the health and nutritional status of Americans (24). The NHANES interview covers demographic, health-related questions, dietary interviews, physical, and laboratory examinations (25). Our study data were obtained from the NHANES database. The data were approved by the National Center for Health Statistics (NCHS) Institutional Review Board (IRB). The NCHS IRB approval and documented consent were obtained from participants. The NCHS IRB Protocol Number is Protocol #98-12. The data used to support the findings of this study from the NHANES website (http://www.cdc.gov/nchs/nhanes.htm).

A total of 15,332 adults (20–85 years) with a questionnaire survey on severe headache or migraine were investigated from NHANES 1999–2004. We excluded pregnant women (*n* = 833) and participants with missing essential information on their demographic (*n* = 526), dietary interview (*n* = 1,710), laboratory examination (*n* = 3,530) or questionnaire survey (*n* = 853). Therefore, 7,880 adults (3,575 men and 4,305 women) were included in our study.

### Headache Classification

We considered participants who answered “yes” to the question: “During the past 3 month, did you have severe headaches or migraines?” to be headache sufferers or migraineurs. The NHANES did not provide additional information about headache or migraine. However, it is reasonable to assume that most of the participants with severe headache suffered from migraine. The American migraine prevalence and prevention (AMPP) study indicated that of the 17.4% of participants who reported “severe headache,” 11.8% met the International Headache Disorder type ii (ICHD-II) migraine criteria, 4.6% met the criteria for “possible migraine,” and only 1% were classified as “other severe headache” (26).

### Dietary Iron and Serum Ferritin Assessment

Dietary iron intake was assessed by a 24-h recall survey. The survey is a retrospective dietary assessment method that provides detailed information on all foods and beverages during a 24-h period (27). Interviews were conducted in-person by trained dietary interviewers. Participants were provided with a standard set of measurement guidelines to help them report the amount and size of food. NHANES used the Food Intake Analysis System (FIAS), and the United States Department of Agriculture (USDA) survey nutrient database to encode access data and convert it into total nutrient intake (28). Detailed dietary survey methods were provided in The NHANES Dietary Interviewers Procedure Manuals (29). In our study, all participants performed the first 24-h dietary recall from 1999 to 2004. In 2003–2004, a second 24-h dietary recall was conducted *via* telephone ~3–10 days after the first recall survey. Due to the lack of information from the second dietary survey (1999–2002), we assessed this based on the first 24-h dietary recall. In addition, we grouped participants according to their dietary iron intake. Participants answered fasting questionnaires to determine whether they were fasting and were interviewed by researchers to determine their suitability for venipuncture. Blood samples were collected at the mobile examination center throughout the day. The blood samples were placed at room temperature for 30–60 min and centrifuged at 2,000 °C for 10 min to obtain the serum. The principle of determination of serum ferritin was immuno-turbidimetry using Roche kits on the Hitachi 912 clinical analyzer. We also grouped participants according to their serum ferritin levels.

### Potential Covariates

Demographic covariates included age, sex, race/ethnicity, education level, and marital status, which were obtained by self-report during the interview; health-related covariates included smoking status, drinking status, physical activity, and BMI; dietary-related covariates included energy intake, protein intake, and carbohydrate intake; clinical-related covariates included hypertension (defined as systolic blood pressure ≥140 mm Hg or diastolic blood pressure ≥90 mm Hg and obtained from self-report), diabetes (obtained from self-report), serum iron, hemoglobin, total cholesterol, and C reactive protein; medication covariates included oral estrogen and birth control pills.

### Statistical Analyses

All analyses incorporated sample weights, stratification, and clustering to account for complex sampling designs following the NHANES analytic guidelines (30). The NHANES weights are calculated every 2 years, but 4-year weights for the 1999–2002 period are available. The data for 1999–2004 cover three 2-year sampling cycles. According to NHANES guidelines, we calculated 6-year weights = 2/3 of the 1999–2002 weight or 1/3 of the 2003–2004 weight. Means and standard deviations or counts and frequencies were described for the continuous or categorical variables, respectively. The Student *t*-test was used for comparisons of continuous variables. The Chi-square test was used to evaluate the differences between categorical variables. Logistic regression models were used to estimate the odds ratios (ORs) and 95% confidence intervals (CIs) of the association between dietary iron intake and serum ferritin and severe headache or migraine, with the lowest quintile of dietary iron intake, and serum ferritin as reference. Furthermore, we stratified analyzing these associations by sex and age, considering the differences in physiological mechanisms of iron metabolism and dietary iron RDA among different genders and age categories.

Restricted cubic spline (RCS) regression (31) was used to flexibly model the association of dietary iron intake and serum ferritin with severe headache or migraine. The reference values were the RDA of dietary iron and the median of serum ferritin, and knots were placed at the 5th, 25th, 50th, 75th, and 95th percentiles. Statistical analyses were performed with R software version 3.2.5 (using packages *Hmisc* and *rms* for RCS). *P* values < 0.05 (two-sided) were considered statistically significant. We adjusted *P* values for multiple comparisons using the Bonferroni correction method.

## Results

### Basic Characteristics

Table 1 shows the basic characteristics of the 7,880 participants, of whom 1,702 (21.6%) had severe headache or migraine. Compared with the participants without headache, the participants with headache were more likely to be younger, female, and living alone, had a lower education level, a lower BMI, a lower dietary protein intake, a lower dietary iron intake, a lower cholesterol level, a lower hemoglobin level, a lower serum iron level, a lower serum ferritin level, and more likely to take birth control pills. The participants with headache or migraine were not likely to be non-Hispanic White, current drinking, former smoking, and hypertension.

**Table 1:** Characteristics of participants with or without headache.

**Characteristic**	**Non-headache**	**Headache**	***p* value**
Participants, No.	6,178	1,702	–
Age, Mean (SD), years	51 (18.5)	44 (15.1)	<0.001
Sex			
Male	3,079 (49.8)	496 (29.1)	<0.001
Female	3,099 (50.2)	1,206 (70.9)	
Race/ethnicity No. (%)			
Non-Hispanic white	3,824 (53.2)	792 (46.5)	<0.001
Non-Hispanic black	1,107 (17.9)	362 (21.3)	
Mexican American	1,312 (21.2)	382 (22.4)	
Others	475 (7.7)	166 (9.8)	
Marital status No. (%)			
Married or living with partner	3,898 (63.1)	1,028 (60.4)	0.042
Living alone	2,280 (36.9)	674 (39.6)	
Education level No. (%)			
< High school	1,844 (29.8)	586 (34.4)	<0.001
High school or GED	1,431 (23.2)	405 (23.8)	
>High school	2,903 (47.0)	711 (41.8)	
Drinking No. (%)			
Never	860 (13.9)	277 (16.3)	<0.001
Current	4,335 (70.2)	1,074 (63.1)	
Former	983 (15.9)	351 (20.6)	
Smoking No. (%)			
Never	3,145 (50.9)	889 (52.2)	<0.001
Current	1,296 (21.0)	475 (27.9)	
Former	1,737 (28.1)	338 (19.9)	
Physical activity No. (%)	1,389 (22.5)	365 (21.4)	0.473
Hypertension No. (%)	1,835 (29.7)	397 (23.3)	<0.001
Diabetes No. (%)	591 (9.6)	158 (9.3)	0.724
BMI, Mean (SD), kg/m^2^	28.3 (6.67)	27.6 (5.87)	<0.001
Energy (kcal/day)	2,089 (988)	2,060 (1029)	0.218
Protein intake (g/day)	78.8 (41.4)	76.0 (44.6)	0.014
Carbohydrate intake (g/day)	258 (128)	262 (136)	0.329
Iron intake (mg/day)	13.4 (7.17)	12.0 (7.05)	<0.001
Total cholesterol (mmol/L)	5.27 (1.10)	5.19 (1.05)	0.012
C reactive protein (mg/dl)	0.22 (0.12)	0.27 (0.11)	0.051
Hemoglobin (g/dl)	14.4 (1.48)	14.0 (1.52)	<0.001
Serum iron (μg/dl)	88.7 (37.5)	84.8 (38.9)	<0.001
Ferritin (μg/L)	88.0 (20.5)	56.9 (15.5)	<0.001
Taking birth control pills No. (%)	1,401 (22.7)	608 (35.7)	<0.001
Use of oral estrogen No. (%)	525 (8.5)	167 (9.8)	0.090

*SD, standard deviation; BMI, body mass index; GED, general educational development. Continuous variables were shown as mean ± SD, and categories variables were shown as percentage*.

### Differences Between Daily Iron Intake and Recommended Dietary Allowance

The recommended dietary allowance (RDA) is an estimate of the average daily intake sufficient to meet the nutritional needs of nearly all healthy individuals of a particular sex and age (32). For US adults, women over 50 years old and all men, the RDA for iron was 8 mg/day. The RDA for iron was 18 mg/day for women aged 20–50 years (33). As shown in Table 2, the average daily iron intake for men and women aged over 50 years, was significantly higher than their RDAs. Only women aged 20–50 years had very lower daily iron intake than their RDAs.

**Table 2:** Dietary iron intake among US adults (≥20 years) in NHANES 1999–2004.

**Age (years)**	**RDAs for iron (mg/day)**	**Iron Intake (mg/day)**	***P* value**
Male 20–50	8	15.8 (8.76)	<0.001
Male over 50	8	14.2 (7.71)	<0.001
Female 20–50	18	11.8 (6.79)	<0.001
Female over 50	8	11.0 (6.31)	<0.001

*RDA, recommended dietary allowances*.

### Association Between Dietary Iron and Severe Headache or Migraine

In the multivariable logistic regression, after adjusting for the possible confounders, the OR for the association between dietary iron intake and severe headache or migraine was 0.721 (95% CI, 0.565–0.920) comparing the highest quintile of iron intake (≥19.94 mg/day) with the lowest quintile of iron intake (≤ 8.20 mg/day) (Table 3).

**Table 3:** Association between dietary iron intake and serum ferritin and headache.

	**OR (95% CI)**						
	**No**.	**Crude**	***P*_**value**_**	**Model 1^**a**^**	***P*_**value**_**	**Model 2^**b**^**	***P*_**value**_**
Dietary iron (mg/day)							
Q1 (≤ 8.20)	1,577	1.00 (reference)		1.00 (reference)		1.00 (reference)	
Q2 (8.21–11.18)	1,584	0.900 (0.765–1.059)	0.204	0.900 (0.762–1.064)	0.219	0.887 (0.774–1.057)	0.179
Q3 (11.19–14.52)	1,567	0.782 (0.662–0.923)	0.004	0.784 (0.660–0.931)	0.005	0.766 (0.634–0.925)	0.006
Q4 (14.53–19.93)	1,577	0.688 (0.581–0.816)	<0.001	0.740 (0.621–0.883)	0.001	0.699 (0.569–0.858)	0.001
Q5 (≥19.94)	1,575	0.701 (0.592–0.830)	<0.001	0.825 (0.690–0.986)	0.035	0.721 (0.565–0.920)	0.009
*P* for trend	–	<0.001	–	0.007	–	0.008	–
Serum ferritin (μg/L)							
Q1 (≤ 30)	1,634	1.00 (reference)		1.00 (reference)		1.00 (reference)	
Q2 (31–61)	1,521	0.765 (0.654–0.894)	0.001	0.932 (0.793–1.094)	0.388	0.921 (0.781–1.087)	0.331
Q3 (62–108)	1,599	0.603 (0.514–0.707)	<0.001	0.919 (0.775–1.090)	0.333	0.883 (0.740–1.054)	0.169
Q4 (109–195)	1,557	0.434 (0.366–0.515)	<0.001	0.796 (0.657–0.964)	0.020	0.735 (0.602–0.897)	0.002
Q5 (≥196)	1,569	0.373 (0.312–0.444)	<0.001	0.791 (0.644–0.971)	0.025	0.706 (0.570–0.875)	0.001
*P* for trend	–	<0.001	–	0.034	–	0.008	–

*OR, odds ratio; CI, confidence interval*.

a *Model 1 was adjusted for age, sex*.

b *Model 2 was adjusted for age, sex, race/ethnicity, smoking, drinking, marital, education level, physical activity, BMI, energy intake, protein intake, carbohydrate intake, hypertension, diabetes, C reactive protein, total cholesterol, serum iron, hemoglobin, oral estrogen and birth control pills*.

After stratifying by age for both sexes, we found that dietary iron was negatively associated with migraine in women aged 20–50 years. The ORs (95% CI) for the association between dietary iron intake and migraine was 0.694 (0.518–0.929), 0.700 (0.511–0.958), and 0.571 (0.398–0.819) comparing Q3, Q4, and Q5 with Q1. For women aged over 50 years and men, there was no significant relationship between dietary iron intake and severe headache or migraine (Table 4).

**Table 4:** Effect of dietary iron on headache in different sex and age groups.

	**OR (95% CI)**		
	**No**.	**Ajusted^**a**^**	***P*_**value**_**
Women 20–50 (years)			
Q1 (≤ 7.77)	490	1.00 (reference)	
Q2 (7.78–10.40)	490	0.841 (0.641, 1.104)	0.212
Q3 (10.41–13.29)	489	0.694 (0.518, 0.929)	0.014
Q4 (13.30–17.47)	490	0.700 (0.511, 0.958)	0.026
Q5 (≥17.48)	489	0.571 (0.398, 0.819)	0.002
*P* for trend	–	0.034	–
Women over 50 (years)			
Q1 (≤ 7.12)	372	1.00 (reference)	–
Q2 (7.13–9.73)	371	0.845 (0.577, 1.238)	0.388
Q3 (9.74–12.44)	372	0.836 (0.555, 1.257)	0.389
Q4 (12.45–16.42)	373	0.659 (0.418, 1.039)	0.072
Q5 (≥16.43)	369	0.615 (0.373, 1.014)	0.057
*P* for trend	–	0.380	–
Men 20–50 (years)			
Q1 (≤ 10.06)	366	1.00 (reference)	–
Q2 (10.07–13.88)	368	0.731 (0.589, 1.009)	0.061
Q3 (13.89–18.01)	363	0.739 (0.497, 1.097)	0.134
Q4 (18.02–24.10)	366	0.968 (0.664, 1.410)	0.864
Q5 (≥24.11)	365	1.060 (0.729, 1.541)	0.761
*P* for trend	–	0.471	–
Men over 50 (years)			
Q1 (≤ 8.97)	350	1.00 (reference)	–
Q2 (8.98–12.12)	350	1.134 (0.677, 1.602)	0.632
Q3 (12.13–15.86)	349	1.022 (0.589, 1.571)	0.939
Q4 (15.87–21.72)	349	0.913 (0.499, 1.472)	0.768
Q5 (≥21.73)	349	0.925 (0.469, 1.521)	0.820
*P* for trend	–	0.936	–

a *The model was adjusted for age, sex, race/ethnicity, smoking, drinking, marital, education level, physical activity, BMI, energy intake, protein intake, carbohydrate intake, hypertension, diabetes, C reactive protein, total cholesterol, serum iron, hemoglobin, oral estrogen and birth control pills*.

**Figure 1:**
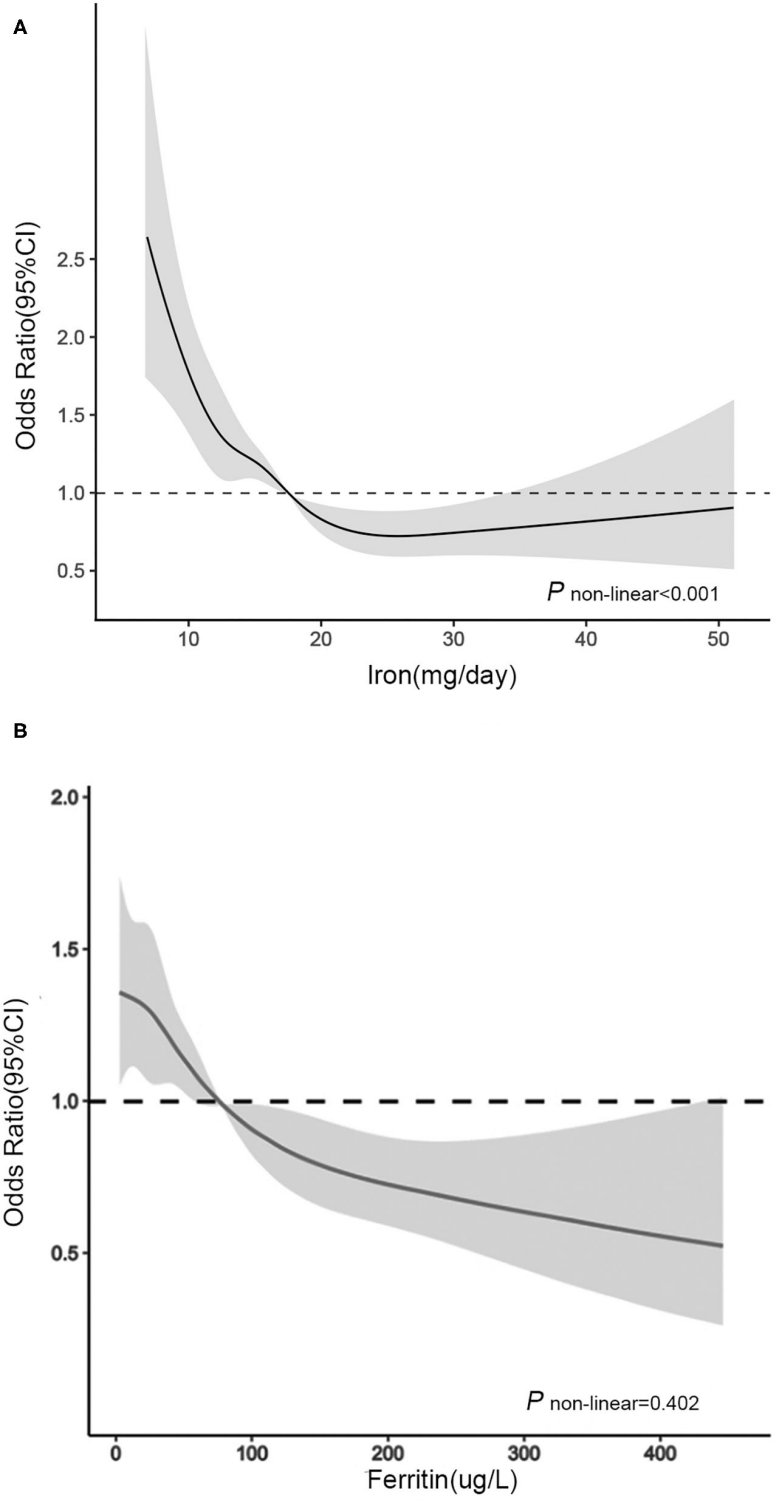
Association between **(A)** dietary iron intake and **(B)** serum ferritin and headache in women of different ages in RCS. The model was adjusted for race/ethnicity, smoking, drinking, marital, education level, physical activity, BMI, energy intake, protein intake, carbohydrate intake, hypertension, diabetes, C reactive protein, total cholesterol, serum iron, hemoglobin, oral estrogen and birth control pills. Solid line, OR; shade, 95% CI.

In RCS (Figure 1A), for women aged 20–50 years, we used the RDA of iron intake (18.00 mg/day) as the reference point and found a non-linear association between iron intake (continuously measured) and severe headache or migraine (*P* < 0.001). We observed that the prevalence of severe headache or migraine decreased with increasing iron intake until iron intake reached ~25 mg/day, after which the risk of severe headache or migraine reached a plateau.

### Association Between Serum Ferritin and Severe Headache or Migraine

We found that serum ferritin was negatively associated with migraine. Compared with the lowest quintile of serum ferritin, the adjusted OR in the highest quintile of serum ferritin was 0.706 (95% CI, 0.570–0.875) (Table 3). After further stratifying by age for both sexes, for women aged over 50 years, the ORs (95% CI) of Q3, Q4, and Q5 were 0.686 (0.475, 0.990), 0.654 (0.452, 0.947), and 0.492 (0.332, 0.730) compared with Q1 (Table 5). The correlation between dietary iron and serum ferritin was extremely weak (*r* = 0.06; *P* < 0.001) (data are not shown).

**Table 5:** Effect of serum ferritin on headache in different sex and age groups.

	**OR (95% CI)**		
	**No**.	**Ajusted^**a**^**	***P*_**value**_**
Women 20–50 (years)			
Q1 (≤ 15)	519	1.00 (reference)	–
Q2 (16–26)	467	1.041 (0.793, 1.367)	0.770
Q3 (27–42)	513	0.922 (0.704, 1.208)	0.557
Q4 (43–70)	472	0.979 (0.742, 1.292)	0.882
Q5 (≥71)	477	0.986 (0.798, 1.362)	0.839
*P* for trend	–	0.909	–
Women over 50 (years)			
Q1 (≤ 34)	378	1.00 (reference)	–
Q2 (35–61)	365	0.834 (0.581, 1.198)	0.326
Q3 (62–97)	375	0.686 (0.475, 0.990)	0.044
Q4 (98–160)	370	0.654 (0.452, 0.947)	0.025
Q5 (≥161)	369	0.492 (0.332, 0.730)	<0.001
*P* for trend	–	0.007	–
Men 20–50 (years)			
Q1 (≤ 77)	368	1.00 (reference)	–
Q2 (78–118)	366	0.848 (0.578, 1.245)	0.401
Q3 (119–168)	363	0.806 (0.546, 1.190)	0.278
Q4 (169–249)	371	0.831 (0.565, 1.223)	0.347
Q5 (≥250)	360	0.706 (0.471, 1.057)	0.091
*P* for trend	–	0.574	–
Men over 50 (years)			
Q1 (≤ 63)	351	1.00 (reference)	–
Q2 (64–113)	349	0.751 (0.461, 1.223)	0.249
Q3 (114–181)	353	0.632 (0.378, 1.057)	0.080
Q4 (182–298)	347	0.642 (0.388, 1.064)	0.086
Q5 (≥299)	347	0.720 (0.438, 1.185)	0.196
*P* for trend	–	0.465	–

a *The model was adjusted for age, sex, race/ethnicity, smoking, drinking, marital, education level, physical activity, BMI, energy intake, protein intake, carbohydrate intake, hypertension, diabetes, C reactive protein, total cholesterol, serum iron, hemoglobin, oral estrogen and birth control pills*.

In RCS (Figure 1B), for women aged over 50 years, we used the median serum ferritin (76 μg/L) as the reference point and found a linear association between serum ferritin (continuously measured), and migraine (*P* = 0.402). The ORs for the association between serum ferritin and migraine were decreased with elevated serum ferritin levels. When the serum ferritin concentration was higher than 76 μg/L, the OR was significantly lower than 1.00.

## Discussion

In these nationally representative cross-sectional studies of US adults, we observed an inverse association between dietary iron and serum ferritin and severe headache or migraine, independent of significant confounders. After stratified analyses by sex and age, we found that dietary iron was negatively associated with migraine in women aged 20–50 years. For women aged over 50 years and men, the relationship between dietary iron intake and migraine was not statistically significant.

The average dietary iron intake (11.82 mg/day) of women aged 20–50 was much lower than their RDA (18 mg/day). The lower dietary iron intake cannot meet the iron needs of young women. Meanwhile, young women had lower serum ferritin levels and lower iron stores. However, for women aged over 50 and men, the average dietary iron intake was higher than their RDAs, and they also had higher serum ferritin levels and higher iron stores. Studies have shown that the absorption of iron is closely related to whether there is sufficient iron in the body, and iron absorption increases when there is insufficient iron storage and decreases when there is sufficient iron storage (34–36). Susan et al. found that the lower the ferritin level, the higher the iron absorption (37). Therefore, women aged 20–50 years may have higher iron absorption than men and women over 50 years, which may be the reason why dietary iron was associated with migraine only in women aged 20–50 years.

Serum ferritin is an important biomarker of body iron storage and a reliable indicator of body iron metabolism (16). Interestingly, we found that serum ferritin was inversely correlated with severe headache or migraine in women aged 50 and above. However, there was no significant relationship between serum ferritin and severe headache or migraine in women aged 20–50 years. Our results found that the mean serum ferritin level in women aged 20–50 years (49.27 μg/L) was much lower than that in women aged 50, and above (82.68 μg/L). We hypothesized that serum ferritin would reduce the prevalence of migraine in women only if it reached a certain threshold, which may be the reason why serum ferritin was not associated with migraine in women aged 20–50 years. Previous studies have shown a decrease in the prevalence of migraine in postmenopausal women, which we believe may be related to increased serum ferritin. Some researchers believe that estrogen may affect the incidence of migraine in postmenopausal women (38, 39). However, after we adjusted for estrogen supplements, the negative association between serum ferritin, and migraine remained in women aged 50 and above. For men, the prevalence of migraine tended to decrease with increasing serum ferritin levels, but it was not statistically significant. Men had lower demand for iron than women, and ferritin levels in the body of men had been relatively stable, so the effect of ferritin on men may not be as significant as that on women.

Several studies have supported our results. Yücel et al. (14) suggested that iron deficiency may affect the pathophysiology of migraine in patients with restless leg syndrome. Patients with iron-deficiency anemia had a high frequency of migraine (15). A case-control study found that female migraineurs had low serum ferritin levels (17). However, the case-control study did not perform further age-stratified analysis, which was different from our study. Iron is an essential trace element for the synthesis of dopamine (18), which plays a role in the pathogenesis of migraine (19, 20). Some studies have shown that iron deficiency could cause dopaminergic neurodegeneration (21–23). We speculate that regulation of dopamine by iron may be a potential mechanism for the beneficial effect of iron against migraine. These findings suggest that iron may play an important role in migraine.

However, Domínguez et al. (40) found increased iron accumulation in some areas of the brain in patients with chronic migraine. Although it has been shown that dietary iron may not cause iron overload (38), we suggest that dietary iron supplements should be adequate to meet the needs of the body and should not be excessive. We found that most women aged 20–50 years consumed less dietary iron than their RDA, therefore, we recommend that women aged 20–50 years should increase awareness of RDA, and increase dietary iron intake. However, we do not recommend that people with low iron needs and sufficient body iron levels, such as women over 50 years and men, take excessive dietary iron.

Our study has several strengths. First, to our knowledge, this is the first and largest nationally representative sample to assess the relationship between dietary iron intake, and migraine in US adults. Second, we further assessed the dose-response effects of dietary iron intake, and serum ferritin on migraine, providing more practical suggestions. There were also some limitations. First, the outcomes of this study, severe headache and migraine, were based on self-reports. We could not distinguish the type of severe headache. But given that the AMPP study classified most severe headache as migraine (26), and it is reasonable to believe that the majority of severe headache reported in this study were migraine. In future studies, defining diagnostic criteria for headache and conducting subgroup analyses based on different headache types will help to better identify populations that benefit from dietary iron and ferritin. Second, dietary data were based on 24-h recalls, a method with inherent limitations in the reliability and validity of nutritional assessments. However, studies have shown that 24-h recalls may provide more details about the type, and quantity of food than food frequency surveys (41, 42). Third, we lack information on perimenopause. Future studies should consider the effect of perimenopause to further investigate the association between iron and migraine. Finally, our study was a cross-sectional study, and no causal inferences can be made. Therefore, further prospective longitudinal investigations are needed to elucidate the causal relationship between dietary iron intake, and serum ferritin and migraine.

## Conclusions

Our study first investigates the association between dietary iron and serum ferritin and severe headache or migraine among American adults. Our observations suggest that dietary iron and serum ferritin may play a role in severe headache or migraine prevention. The RDA of iron, originally designed for general nutrition in women aged 20–50 years, and was found to also be suitable for preventing migraine in our study. We recommend that women aged 20–50 years should have higher awareness of RDA and increase their dietary iron intake if needed.

## Data Availability Statement

The raw data supporting the conclusions of this article will be made available by the authors, without undue reservation.

## Ethics Statement

The studies involving human participants were reviewed and approved by Institutional Review Board (IRB). The patients/participants provided their written informed consent to participate in this study.

## Author Contributions

S-HM and Y-SZ designed research and wrote the manuscript. S-HM, H-BZ, and XL conducted research and analyzed the data. All authors contributed to the acquisition or interpretation of the data and approved the final manuscript.

## Conflict of Interest

The authors declare that the research was conducted in the absence of any commercial or financial relationships that could be construed as a potential conflict of interest.
